# Metabolic Factors and Chronic Hepatitis C: A Complex Interplay

**DOI:** 10.1155/2013/564645

**Published:** 2013-07-17

**Authors:** Fabio Salvatore Macaluso, Marcello Maida, Maria Giovanna Minissale, Teresa Li Vigni, Simona Attardo, Emanuele Orlando, Salvatore Petta

**Affiliations:** Section of Gastroenterology, DiBiMIS, University of Palermo, Piazza delle Cliniche 2, 90127 Palermo, Italy

## Abstract

In the last years, several lines of evidence showed how metabolic factors may influence the natural history of patients with chronic hepatitis C. Chronic HCV infection is able to perturb the metabolic homeostasis of the host, in a context of complex interactions where pre-existent metabolic status and genetic background play an important role, allowing us to state that HCV infection is a systemic disease. In this review, we discuss the most recent lines of evidence on the main metabolic factors that are known to be associated with CHC, namely, insulin resistance/type 2 diabetes, steatosis, visceral obesity, atherosclerosis, vitamin D, menopause, fructose and coffee intake, lipoproteins, methylenetetrahydrofolate reductase status, and hyperuricaemia. In particular, we focus on the pathophysiological mechanisms underlying the correlation between HCV infection and metabolic disorders, the impact of metabolic factors on the progression of liver and non-liver-related diseases, and, on the contrary, the possible influence of chronic HCV infection on metabolic features. In this setting, the importance of a multifaceted evaluation of CHC patients and a prompt correction of modifiable metabolic risk factors should be emphasized.

## 1. Introduction

**Figure 1:**
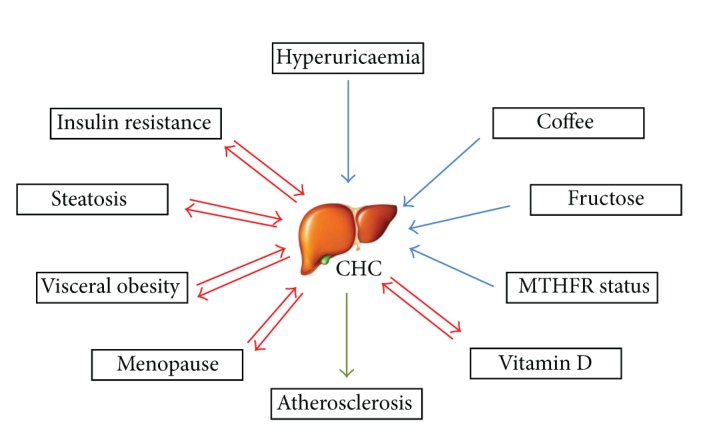
The interplay between metabolic factors and chronic HCV infection.

Hepatitis C virus (HCV) infection is one of the main causes of chronic liver disease worldwide, and it has reached a pandemic spread [1]. The risk of progression from chronic HCV infection to cirrhosis and its clinical outcomes is highly variable, and many factors are believed to accelerate disease progression and to impact the likelihood of sustained virological response (SVR) after pegylated interferon/ribavirin therapy. In this line, a considerable amount of evidence showed how metabolic factors may influence the natural history of patients with chronic hepatitis C (CHC) and affect the outcome of antiviral therapies, particularly insulin resistance (IR) and steatosis [2]. This correlation is not unidirectional [3], since chronic HCV infection is able to influence glucose and lipid metabolism and thus to perturb the metabolic homeostasis of the host leading to extrahepatic consequences (Figure 1). The result of this complex interaction is the conceptual translation of CHC from a localized to a systemic disease and, therefore, the need for the clinician to evaluate a patient with CHC focusing on liver disease and on associated metabolic conditions. 

We discussed the most recent lines of evidence on the role of the main metabolic factors that are known to be associated with CHC, the pathophysiological mechanisms underlying the correlation between HCV infection and metabolic disorders, the impact of metabolic factors on the progression of liver and liver-related diseases, and, on the contrary, the possible influence of chronic HCV infection on metabolic features. 

## 2. Insulin Resistance

IR is defined as the condition in which higher insulin concentrations are required to achieve a normal metabolic response, otherwise not reached by normal insulin levels, and wherein the alterations mainly belong to postreceptorial transduction mechanisms [4]. IR is correctly regarded as the pathophysiological keystone of metabolic syndrome (MS), which represents a major cause of morbidity and mortality and whose prevalence is increasing worldwide [5]. Given the high spreads of MS and CHC, the chances of coexistence of these two conditions in a single patient are elevated. Nevertheless, this overlap is not simply coincidental: in subjects with CHC, the presence and severity of IR are related to host factors, mostly visceral obesity [6], but in addition, many experimental [7] and clinical [8] studies suggested how HCV infection itself seems to be able to perturb glucidic homeostasis, leading to hepatic and extrahepatic IR. In this line, several lines of evidence are currently available on the ability of HCV to induce IR. Cross-sectional studies showed that the prevalence of diabetes in patients with CHC is superior to the one reported in other cohorts, such as subjects with other chronic liver diseases, human immunodeficiency virus-infected patients or drug users [9]. IR, evaluated through the homeostasis model assessment (HOMA-IR), is associated with HCV genotypes 1 and 4 and their viral load, which is higher than that in matched patients with chronic hepatitis B [8], is increased even at early stages of liver fibrosis [10] and, finally, may decrease across followup after SVR achievement [11]. In patients with CHC, mechanisms of IR can be found not only in liver, where they are expressed by an increased endogenous glucose production, but also in muscle tissue, resulting in a reduced glucose uptake in muscle [12]. Conversely, glucidic function of adipose tissue is not affected in HCV-associated IR, unlike what is commonly described in the course of “pure” IR conditions. This has prompted speculation that HCV may be able to affect insulin signalling inducing both hepatic and peripheral IR [3] via several direct or indirect mechanisms (downregulation of suppressor of cytokine signalling (SOCS) and protein phosphatase PPA2, upregulation of tumour necrosis factor-*α* and induction of proinflammatory cytokines or other unknown soluble mediators). Excellent reviews described molecular pathways of HCV-induced IR [13, 14].

The relevance of IR in patients with CHC appears of great interest considering its potential influence on severity and progression of chronic liver disease, where IR can act both directly and indirectly, by inducing steatosis. A large population-based study [15] on patients with chronic liver disease of various etiology showed that IR and T2D were independent predictors of overall mortality, with the remarkable exception of CHC patients; nevertheless, both T2D and IR were independently associated with liver-related mortality in patients with CHC. In this line, both cross-sectional and prospective studies repeatedly highlighted how IR and/or steatosis are associated with the severity and the progression of hepatic fibrosis and thus with the clinical progression of liver disease [16, 17]. While steatosis acts via collagenous deposition, generation of lipid peroxides, and finally stellate cell activation [18], IR seems to be a powerful promoter of fibrogenesis via direct hepatic stellate cell stimulation, tumor necrosis factor-*α* and connective growth factor production, and ductular reactions induction [19]. In addition, several studies also suggested that full-blown T2D may further increase fibrogenesis and thus the risk of severe fibrosis in a context of IR [6]. This latter observation has been recently questioned by an interesting retrospective paper by Giordanino and colleagues [20]: even if diabetic patients had a higher number of diabetes- and liver-related events and higher mortality than nondiabetics, diabetes was not an independent factor for liver-related events, and diabetes-related events were lower in the HCV-positive group. Nevertheless, considering the high prevalence of cirrhosis and non-SVR among diabetic CHC patients, these data could however suggest an indirect role of diabetes in liver disease progression and in lack of SVR [2]. Finally, IR was found to be associated with the presence ofoesophageal varices in patients with HCV-related compensated cirrhosis [21]. This suggests the ability of insulin to modulate dynamic components of portal hypertension, for example, the endothelial synthesis of nitric oxide and endothelin [22, 23], other than induce architectural disturbances through the promotion of fibrogenesis. 

As obesity and T2D are well-known risk factors for the development of many types of cancer, many experimental and observational studies on a potential increase of hepatocellular carcinoma (HCC) development in patients with CHC and IR were performed. A population-based study [24] revealed that the presence of T2D was associated with a 3-fold increase risk of HCC occurrence and that this risk was higher in patients with both HCV and T2D and even higher in presence of obesity. In addition, in a recent prospective study by Nkontchou et al., IR itself was an independent risk factor for HCC development in HCV-related cirrhosis and, at the same time, a predictor of liver-related death or transplantation [25]. A vast literature exists on potential molecular mechanisms and mediators underlying the processes of liver carcinogenesis, including IR and hyperinsulinaemia, oxidative stress, and imbalances between proinflammatory and anti-inflammatory cytokines, even if further data are needed [26]. 

While lines of evidence supporting the association between IR and progression of CHC appear overall to be solid, controversial data exist on the role of IR as a predictor of both rapid (RVR) and SVR in CHC patients treated with pegylated interferon/ribavirin. Recent studies in European [27], Caucasian-American, and African-American [28] patients infected with genotype 1 HCV showed that IR is associated with a lower likelihood to achieve an SVR after antiviral treatment with peg-interferon and ribavirin. The same results were recorded for genotypes 2, 3 [29], and 4 [30]. Such data have been emphasized by a recent meta-analysis [31], which quantified in a difference approximately equal to 20% the likelihood of SVR between patients with and without IR. In addition, lower HOMA-IR levels have been associated with RVR achievement in non-diabetic, noncirrhotic genotype 1 HCV patients, thus suggesting a relevant role of IR in the early phase of viral kinetics [32]. The roots of these phenomena may be sought in the mutual interference between insulin and interferon signalling via SOCS-3 liver expression [33]. Nevertheless, other authors [34–36] did not observe an association between IR and SVR, showing that only moderate/severe steatosis, but not IR, was negatively associated with SVR. Overall, these observations may lead to hypothesize that both IR and steatosis could impact the likelihood of SVR and that the different results among studies may be related to pre-existent differences in metabolic dysfunctions and genetic backgrounds in the examined cohorts. In this line, interesting findings emerged through analysis of the interaction between interleukin-28B (IL-28B) genotype, an important host factor for SVR achievement after antiviral therapy, and IR. In white patients with genotype 1 CHC, the IL28B rs12979860 CC genotype was associated with reduced IR, and the same polymorphism and IR were associated with SVR at multivariate analysis, but not steatosis [37]. Finally, new data are currently emerging on the impact of IR on new direct acting antiviral-based treatments. In naïve genotype 1 CHC patients, baseline HOMA-IR was not correlated with virological response to telaprevir-based therapy, even if SVR was associated with improved HOMA-IR [38]; in experienced patients, baseline HOMA-IR correlated with SVR in univariate but not multivariate analysis [39]. Even if these results could suggest that HOMA-IR may not have a direct causal relationship with virological response to telaprevir-based therapy, caution is warranted because data analysis did not take into account the role of IL28B polymorphisms and steatosis (assessed in a fraction only of naïve patients and not evaluated in experienced subjects).

## 3. Steatosis

Steatosis is an extremely common histological finding in patients with CHC, with a prevalence ranging from 40 to 80% [40], superior to the rate reported in chronic liver diseases of different etiology [41]. This high variability is probably due to a different distribution of known risk factors for steatosis, such as obesity, T2D, alcohol, and dyslipidemia, in the examined cohorts. However, even when these prevalence data are adjusted for metabolic risk factors, the proportion of patients with HCV and intrahepatic fat accumulation remains high (30–40%) [42]. Consequently, although NAFLD and CHC are both common conditions in the general population, the rate of steatosis observed in CHC is 2.5 times bigger than the expected value on the basis of a simple random coexistence [42]. These data suggest that not only host but also viral factors may participate in steatosis induction in CHC. A direct effect of HCV on steatogenesis is particularly relevant in genotype 3 patients, where steatosis is more frequent and severe [43], due to specific genomic sequences of HCV genotype 3 favoring lipids accumulation in the liver. The core protein may be sufficient to induce steatosis, the genotype 3a being the most efficient [44], although sequences outside the core seem to concur [45]. HCV is able to (in)directly promote the intracytoplasmic accumulation of fat in the liver increasing the hepatic synthesis of fatty acids and reducing the mechanisms of secretion and degradation of lipids [46]. Molecular details can be found in excellent reviews [3, 13, 14]. In this line, HCV-induced overexpression of an adipocytokine which takes part in steatogenesis, retinol binding protein 4 (RBP4) [47], has been recently advocated as a possible expression of a virus-linked pathway to steatosis in CHC, largely unrelated to IR [48, 49]. Interestingly, steatosis may decrease after SVR [36] and has been related to HCV viral load [40]. All these events are more noticeable in patients with genotype 3 HCV, in which steatosis has been referred as “viral.” Conversely, in non-3 genotypes infections, steatosis is regarded as “metabolic,” since it seems to be correlated more strictly with age and metabolic variables [50]. Of note, many mechanisms accounting for HCV-related steatosis can also promote IR. On the other hand, patients with high degrees of viral steatosis do not steadily present high levels of IR, and *vice versa*: studies reported that HOMA score is higher in patients with genotypes 1 and 4 HCV [8], while HOMA levels are the lowest in patients with genotype 3 [10]. However, these findings are not univocal and likely dependent on different baseline metabolic features of analyzed cohorts.

The clinical relevance of steatosis in CHC patients lies in the fact that numerous studies have identified in liver fat accumulation a potential risk factor for progression of fibrosis, HCC occurrence, and lower likelihood of SVR achievement after antiviral therapy. Both cross-sectional and prospective papers identified steatosis as a predictor of liver fibrosis [51–53], with a major role related to metabolic rather than virus-induced steatosis, even if data are sometimes discordant. Interestingly, a longitudinal French study showed that worsening of steatosis was the only independent factor associated with the progression of liver fibrosis in untreated patients with CHC [54]. In this line, the presence of steatosis has been related to possible increased oxidative stress and to phenomena of lipid peroxidation [55, 56] which may assist in fibrogenesis promotion. Other works highlighted how steatosis seems correlated with higher levels of proinflammatory cytokines, which are able to activate stellate cells [57, 58]. The same condition of IR has been invoked as a link between steatosis and fibrosis through the capability of insulin, glucose, and leptin, whose receptors are expressed on stellate cells, to induce the production of connective tissue growth factor [59, 60]. A further possible mechanism of steatosis-induced fibrogenesis could be related to the evidence that liver fat accumulation is associated with increased apoptotic cell phenomena, which are able to activate stellate cells [61, 62]. 

About steatosis and carcinogenesis, several clinical papers have repeatedly found an association between fatty liver and HCC development in patients with CHC [63]. Indeed, in vitro and in vivo studies showed that HCV core protein expression promotes liver fat accumulation and, at the same time, may contribute to carcinogenesis [64, 65], even if molecular pathways are not yet fully understood. A persistent activation of PPAR*α* was highlighted in mice models [66], but this observation was not confirmed in HCV-infected humans [67]. Furthermore, many studies reported that hepatic steatosis is negatively correlated with SVR rates after peg-interferon and ribavirin treatment [17, 35]. This association may be explained through mechanisms that involve IR-induced SOCS, which in turn are responsible for a reduced activation of STAT, proteins involved in interferon signalling [68]. This association seems more specific of metabolic steatosis rather than viral one, since steatosis observed in genotype 3 patients has not been related to decreased likelihoods of SVR [36]. Finally, interesting findings relating steatosis and steatosis-induced liver complications with specific single nucleotide polymorphisms are emerging. The patatin-like phospholipase domain-containing 3 (PNPLA3) rs738409 C>G single nucleotide polymorphism is a genetic determinant of liver fat accumulation [69] able to influence fibrosis severity in NAFLD patients [70], and it has been associated with severe steatosis, fibrosis stage, treatment response, and HCC occurrence in subjects with CHC [71]. In genotype 1 CHC patients, CC polymorphism of IL28B was associated with higher levels of total and low-density lipoprotein cholesterol, lower levels of triglycerides, and a lower prevalence of IR and moderate-severe steatosis compared to patients with different genotypes [37].

## 4. Visceral Obesity

Originally considered a simple passive depot for calories storage, visceral adipose tissue is now regarded as an endocrine site producing several substances able to regulate energetic metabolism, immunity, and inflammation and thus to influence the pathogenesis of cardiovascular disease, IR, and diabetes [72, 73]. In addition, visceral adiposity, evaluated through magnetic resonance, has been associated with liver fat accumulation in healthy subjects [74, 75] and with severity of necroinflammation, and fibrosis in patients with NASH [76]. In a CHC setting, an association between visceral obesity, steatosis and fibrosis was initially found using waist circumference and body mass index (BMI) [77, 78], which may be considered surrogate markers of visceral adipose tissue, even if not entirely accurate. In a more precise way, visceral adiposity index (VAI) is a marker of adipose distribution and dysfunction reflecting nonclassic cardiometabolic risk factors such as altered production of adipocytokines/cytokines, increased lipolysis, and plasma-free fatty acids [79] that has been independently associated not only with steatosis but also with necroinflammatory activity, in patients with genotype 1 CHC [35]. This index was also related to viral load, a finding consistent with several papers that have already suggested a direct association between viral load and BMI [80] and between HCV RNA status and obesity [81]. Overall, these aspects may lead to speculate that, on one hand, adipose tissue could offer fatty substrates and a proinflammatory status promoting HCV replication and that, on the other hand, HCV could molecularly interfere with adipocyte function indirectly, by increasing the inflammatory status and, directly, by colonizing adipocytes or immune cells infiltrating adipose tissue. In this line, interesting findings were also derived from the Hepatitis C Antiviral Long-Term Treatment Against Cirrhosis (HALT-C) trial cohort [82]; authors found an association between several weight-related features and increased rates of histological or clinical progression of CHC: not only IR and histologic features of fatty liver disease at baseline but also weight change during the trial were strongly associated with progressive liver disease.

Finally, new data on a potential role of obesity in affecting SVR rates after treatment with protease inhibitors are emerging; a recent paper by Poordad and colleagues highlighted how, in previously untreated patients, baseline predictors of good response after peg-interferon, ribavirin, and boceprevir treatment included not only IL-28B genotype, low baseline viral load, and absence of cirrhosis but also lower BMI; in addition, BMI was associated with interferon responsiveness (defined as ≥1 log10 HCV-RNA decline at week 4) and with SVR adjusted for log10 HCV-RNA decline at week 4 [83]. In this regard, further evidences are urgently needed.

## 5. Atherosclerosis

In view of the complex overlap between CHC and MS and its features, several recent studies aimed to evaluate if an increased risk of atherosclerosis, cardiovascular events, and related mortality in patients with CHC exist. However, the presence of such an association is not as obvious, at least theoretically, if we consider the typical low-risk lipid profile of most patients with CHC. 

In a large population study from Northern Europe, HBV and HCV infections were not associated with an increased risk for cardiovascular events, including carotid atherosclerosis, myocardial infarction, and stroke [84]. Conversely, a recent long-term prospective study revealed that chronic HCV-infected subjects have higher mortality from both hepatic and extrahepatic diseases, with a hazard ratio of 1.50 for circulatory diseases [85]. In this line, other studies [86–90] showed that atherosclerosis, assessed by carotid artery plaques and/or intima-media thickness (IMT), was increased in patients with CHC compared to healthy controls. In a large prospective study, our group recently reported that the prevalence of asymptomatic carotid atherosclerosis is elevated in CHC patients compared to matched controls and highlighted an association between carotid vascular damage and severity of fibrosis [91]. These findings are clearly in line with other surveys showing that clinical diagnosis of HCV infection is per se an independent risk factor for increased carotid IMT [90] and for cerebrovascular deaths [92]. The pathophysiological mechanisms which may explain this correlation are not clear, but it can be speculated that the proinflammatory mechanisms underlying liver fibrogenesis could be systemically activated, promoting atherosclerosis [91]; in addition, experimental lines of evidence have demonstrated the presence of HCV genomic material within carotid plaques in HCV-infected patients, assuming a possible viral direct action [93]. In this line, Adinolfi and colleagues recently observed that also viral load and hepatic steatosis are associated with the presence of carotid atherosclerosis in CHC subjects, thus assuming that HCV infection could be a relevant risk factor for carotid atherosclerosis occurrence via viral load and steatosis [94]. In contrast to these findings, Mostafa and colleagues recently demonstrated that IMT was associated with classical cardiovascular risk factors, such as systolic blood pressure and LDL cholesterol, while HCV infection was not associated [95]; a recent population-based Japanese study showed a paradoxically lower risk of atherosclerosis in CHC patients compared with healthy controls, even if an increased prevalence of IR in patients with HCV infection is confirmed [96]; Younossi and colleagues found that chronic HCV infection is independently associated with the presence of metabolic conditions (IR, T2D and hypertension) and, interestingly, with the presence of congestive heart failure, but not with increased rates of ischaemic heart disease and stroke [97]. Overall, the results of these studies highlight the presence of ambiguous data on the possible association between HCV infection and cardiovascular risk and the need for further studies in order to obtain external validation of these data in different, for example, per se “metabolic” and “nonmetabolic,” populations.

## 6. Vitamin D

Pleiotropic extraskeletal effects of vitamin D are exerted through the modulation of transcription of over 200 genes involved in immune response, inflammation, cell differentiation, and fibrogenesis and have been recently investigated in settings of chronic liver diseases, including CHC [98]. Our group firstly reported that genotype 1 CHC subjects show a higher prevalence of 25-hydroxyvitamin D (25[OH]D) deficiency compared to a matched control population, also in patients with minimal liver damage, and found an independent inverse relationship between 25(OH)D serum levels and severity of liver fibrosis [99]. Even if other papers observed no associations between vitamin D status and fibrosis stage [100, 101], these findings were further confirmed by other authors [102, 103] and supported by experimental studies showing that vitamin D interacts with its nuclear vitamin D receptor protecting against oxidative stress production [104], influencing the migration, proliferation, and gene expression of fibroblasts [105, 106] and reducing the inflammatory and fibrogenic activity of liver stellate cells [107, 108]. In addition, several studies [99–101] reported a correlation between low 25(OH)D levels and higher necroinflammatory activity, a link that has been suggested to be related to a decreased liver expression of the 25-hydroxylase CYP27A1, enzyme involved in vitamin D3 liver hydroxylation, caused by HCV infection itself [99]. In addition, on the basis of a recent genome-wide study which identified genetic variants affecting 25(OH)D serum levels in healthy populations [109], our group recently reported that GG homozygosis for rs12785878 DHCR7 gene (one of the polymorphism linked to lower serum levels of [25(OH)D], near dehydrocholesterol reductase), together with lower 25(OH)D levels, is independently associated with the severity of liver fibrosis in patients with genotype 1 CHC, thus suggesting that DHCR7 genotype could also prompt fibrogenesis by itself via other direct/indirect mechanisms [110].

While the role of vitamin D status in treatment regimens that include protease inhibitors is yet to be studied, its weight has been extensively investigated in dual therapy with peg-interferon plus ribavirin. First, vitamin D serum levels have been related to RVR achievement, thus assuming a role complementary to IL28B polymorphisms in enhancing the correct prediction of SVR [100, 111]. In addition, Vitamin D deficiency has been associated with failures in achieve an SVR after antiviral therapy in genotypes 1 [94, 95], 2, and 3 [103] HCV infection in some cohorts, but not in others ([101, 103] for genotype 1; [100] for genotype 2-3). Interestingly, prospective data from two small randomized clinical trials found that vitamin D3 supplementation improves SVR in genotypes 1, 2, and 3 HCV infections treated with peg-interferon plus ribavirin [112, 113]. Even if these results are sometimes discordant, the rationale for an immunomodulator capability of vitamin D can be found in experimental studies which show the potential ability of vitamin D signalling to interfere with T cells function and immune response [107, 114]. Consequently, further investigations on the relationship between vitamin D supplementation and SVR may be advisable, even in the rapidly evolving era of direct acting antivirals-based therapy.

## 7. Reproductive Status and Menopause

Studies performed on large cohorts have shown that high levels of estrogens, as typically observed during pregnancy [115], are associated with a reduced necroinflammatory activity in HCV-infected women and that the rate of fibrosis progression in CHC is twice as fast in men than in women [116, 117]. Similarly, menopause has been associated with an accelerated liver fibrosis in women with CHC, an event that may be prevented by long-term hormonal replacement therapy [118]. It has been speculated that this aspect may be secondary to menopause-induced alterations in hormonal balance, in particular the reduction of estrogen levels and the decrease of estradiol/testosterone ratio. These changes result in a disequilibrium in proinflammatory and anti-inflammatory cytokines levels with a subsequent increase in necroinflammatory activity and thus faster fibrosis progression [119].

Reproductive status could affect the response to pegylated interferon/ribavirin therapy, even if the relationship between gender and SVR is still controversial. While some papers showed that the percentages of SVR did not differ between men and women [120], other authors have identified in female gender an independent factor for SVR achievement [121]. A recent study showed that, after stratifying the female population for pre- and postmenopausal status, postmenopausal women have similar progression of liver damage and equal SVR rates than in males but lower than in women in reproductive age [122]; in addition, early menopause was the only factor independently associated with lack of SVR in women with genotype 1 chronic HCV infection [122]. This phenomenon may be related to the alterations of inflammatory factors induced by estrogens deprivation, particularly the decrease of hepatic expression of tumor necrosis factor-*α* and of circulating levels of IL-6, imbalances that may interfere with the response to peg-interferon and ribavirin antiviral therapy [122]. In this line, it should be also mentioned that in postmenopausal women peg-interferon *α*-2B plus ribavirin seems to be more effective than peg-interferon *α*-2A plus ribavirin [123]. The reasons for this phenomenon are not completely understood, but different pharmacokinetics of the two peg-interferons may be theorized. In fact, in postmenopausal women there is an increase in body weight and a different fat distribution induced by hormonal changes; in this context, peg-interferon *α*-2A has a predominant distribution in plasma and liver and thus it may have a lower bioavailability compared to peg-interferon *α*-2B, which also distributes within extrahepatic tissues, such as cytokine-producing visceral fat tissue. 

Interestingly, while menopause may affect the progression and outcome of therapy in CHC, chronic HCV infection in turn may influence the postmenopausal status and its clinical presentation. A recent work demonstrated that HCV infection is independently associated with natural menopause, controlling for age, and that HCV women have a higher prevalence of vasomotor symptoms. The mechanisms by which HCV infection impacts on menopause are not clear, but they may be related to an impaired estrogen metabolism in the liver [124].

Surely, more data are needed to better define the reciprocal interaction between menopause and HCV infection and to evaluate the possible influence of reproductive hormonal status on response to new treatments with protease inhibitors and interferon-free regimens. In this line, an ongoing study in naïve and experienced CHC patients is assessing the impact of menopause on a boceprevir-based antiviral therapy.

## 8. Fructose and Coffee Intake, Lipoproteins, Methylenetetrahydrofolate Reductase Status and Hyperuricaemia

During the last decades, dietary fructose intake has increased worldwide [125]. Several studies on mice showed potential deleterious metabolic effects of fructose, including systemic inflammation, increased lipogenesis and worsening of IR, and obesity [126, 127]. In this line, clinical studies observed that a diet rich in fructose is able to reduce insulin sensitivity [128] and to promote obesity in healthy subjects [129, 130], and it is linked to features of MS and to severity of liver fibrosis in patients with NAFLD [131]. Nowadays, there are few data on a potential role of fructose in the progression of CHC. Tyson and colleagues [132] performed a cross-sectional study on HCV-infected male subjects, finding no significant associations between dietary fructose intake and fibrosis risk, assessed by Fibro-SURE Actitest, even if a significant association between a moderate fructose intake (30 to 48 g/d) and severe necroinflammation was observed. Conversely, preliminary data from our group on a cohort of genotype 1 CHC patients (Petta S. et al., unpublished data) show that only industrial, but not fruit fructose, would be associated with liver injury. Obviously, additional evidences are needed to fully clarify the potential role of fructose intake on hepatic inflammation and fibrosis in CHC subjects.

Several past works have linked coffee drinking with a protective role on serum liver function tests, specifically ALT, AST, and gamma-glutamyltransferase levels [133–137]. More recently, in a large prospective cohort derived from of the Hepatitis C Antiviral Long-Term Treatment Against Cirrhosis (HALT-C) trial, regular coffee consumption was a predictor of less severe steatosis on liver biopsy and of lower rates of histological and clinical progression [138]. In addition, an increased consumption of coffee seems to be able to reduce the risk of liver cancer, as stated by a recent meta-analysis [139], and it has been associated with improved SVR rates after peg-interferon plus ribavirin therapy in CHC, even if the pathophysiological pathways through which coffee may influence liver diseases are not fully understood [140].

A number of lines of evidence suggested a relationship between lipoproteins and HCV cell cycle [141]. In particular, it is evident that CHC patients show lower serum low-density lipoproteins (LDL) [142], which in turn are inversely associated with the severity of liver fibrosis [143] and, directly, with the likelihood to achieve RVR and SVR after pegylated interferon/ribavirin therapy [141, 143–146]. These aspects have been related, on one hand, to a competition for LDL receptor sites which prevents viral entry into hepatocytes and thus to an increased exposure of HCV to the host serum immune response [143–146] and, on the other hand, to the association between higher total and LDL- cholesterol levels with the rs12979860 CC IL-28B polymorphism [147]. In this line, also hyperhomocysteinemia and methylenetetrahydrofolate reductase (MTHFR) C677T point mutation have been investigated in order to evaluate their potential role in CHC. Clinical studies found a link between higher homocysteine levels, MTHFR status, and severity of both steatosis and fibrosis progression in patients with CHC [148, 149]. In addition, other works also identified hyperhomocysteinemia as a negative risk factor for SVR achievement after standard antiviral therapy [150]. In this regard, our group reported a remarkable increase of homocysteine serum levels in genotype 1 CHC patients, not related to MTHFR status, and an independent association between MTHFR C677T homozygosis and higher total and LDL-cholesterol levels, a link which could suggest a possible indirect interference of MTHFR status, via modulation of cholesterol levels, on liver fibrosis and response to antiviral therapy [151].

On the basis of several evidences observing an independent relationship between uric acid serum levels and ultrasonographic diagnosis of NAFLD [152–158], histological severity of NAFLD [159] and development over time of cirrhosis or death because of cirrhosis [160], some studies recently investigated uric acid uric serum levels in patients with CHC. Whereas Pellicano and colleagues linked higher acid serum levels to poor responses to peg-interferon and ribavirin-based therapy [161], a recent paper of our group [162] stated that hyperuricaemia is associated with severity of steatosis, even if not directly associated with lower SVR percentages, representing, via steatosis induction, a potential indirect factor affecting liver damage and poor response to therapy. These findings are supported by experimental data which showed that uric acid may be able to induce IR and others important events involved in steatogenesis, such as systemic inflammation, endothelial dysfunction, and oxidative stress [163, 164], but they obviously need to be confirmed through prospective studies. 

## 9. Conclusions

This overview on main metabolic factors associated with CHC allows us to firmly state that HCV infection is a systemic disease, leading to metabolic consequences due to the interaction of HCV with glucose and lipid homeostasis. This results in IR/T2D and steatosis induction and in the other metabolic features previously discussed, and commonly observed in these patients. Overall, metabolic factors strongly affect the natural history not only of chronic liver disease but also of not liver-related diseases, in a context of complex interplays where pre-existent metabolic disorders and genetic backgrounds play a relevant role. 

In the future, some points should be particularly focused on. The suspected role of IR in increasing the rate of cardiovascular events deserves further prospective analysis to rule out confounders, such as coexistent NAFLD; given the potential weight of IR on SVR achievement, lines of evidence on efficacy of insulin sensitizer therapy are inconclusive yet, and the impact of new pharmacological supports, such as STAT-C agents, should be carefully investigated; the clinical relevance of “minor” metabolic factors, such as vitamin D status or visceral obesity, should be further evaluated in prospective studies, especially to assess their impact on the four-week sensitivity to pegylated interferon/ribavirin; finally, single nucleotide polymorphisms of PNPLA and IL28B genes should be further investigated, since they are currently giving a genetical-pathophysiological key to a better understanding of the mechanisms behind the interactions between metabolic dysfunctions, HCV infection, and viral clearance [165]. Anyway, considering the accelerated progression of liver disease and the cardiometabolic risks, two imbricated and equally important aspects in patients with CHC, lifestyle modification, for example, physical activity and healthful diet, seem mandatory.
